# Effects of Annealing on Characteristics of Cu_2_ZnSnSe_4_/CH_3_NH_3_PbI_3_/ZnS/IZO Nanostructures for Enhanced Photovoltaic Solar Cells

**DOI:** 10.3390/nano10030521

**Published:** 2020-03-13

**Authors:** Chzu-Chiang Tseng, Gwomei Wu, Liann-Be Chang, Ming-Jer Jeng, Wu-Shiung Feng, Dave W. Chen, Lung-Chien Chen, Kuan-Lin Lee

**Affiliations:** 1 Institute of Electro-Optical Engineering, Department of Electronic Engineering, Chang Gung University, Taoyuan 333, Taiwan; 2 Chang Gung Memorial Hospital, Keelung 204, Taiwan; 3 Department of Electro-Optical Engineering, National Taipei University of Technology, Taipei 106, Taiwan

**Keywords:** CZTSe, hole-transporting material, perovskite, IZO, magnetron sputtering

## Abstract

This paper presents new photovoltaic solar cells with Cu_2_ZnSnSe_4_/CH_3_NH_3_PbI_3_(MAPbI_3_)/ZnS/IZO/Ag nanostructures on bi-layer Mo/FTO (fluorine-doped tin oxide) glasssubstrates. The hole-transporting layer, active absorber layer, electron-transporting layer, transparent-conductive oxide layer, and top electrode-metal contact layer, were made of Cu_2_ZnSnSe_4_, MAPbI_3_ perovskite, zincsulfide, indium-doped zinc oxide, and silver, respectively. The active absorber MAPbI_3_ perovskite film was deposited on Cu_2_ZnSnSe_4_ hole-transporting layer that has been annealed at different temperatures. TheseCu_2_ZnSnSe_4_ filmsexhibitedthe morphology with increased crystal grain sizesand reduced pinholes, following the increased annealing temperature. When the perovskitefilm thickness was designed at 700 nm, the Cu_2_ZnSnSe_4_ hole-transporting layer was 160 nm, and the IZO (indium-zinc oxide) at 100 nm, and annealed at 650 °C, the experimental results showed significant improvements in the solar cell characteristics. The open-circuit voltage was increased to 1.1 V, the short-circuit current was improved to 20.8 mA/cm^2^, and the device fill factor was elevated to 76.3%. In addition, the device power-conversion efficiency has been improved to 17.4%. The output power *P_max_* was as good as 1.74 mW and the device series-resistance was 17.1 Ω.

## 1. Introduction

Photovoltaic (PV) devices provide electrical energy directly from sunlight, and have been one of the promising technologies in the renewable energy industry. The further improvement in the efficiency and reliability and also reduction in cost are in great demand, for the healthy development of global economy. The organic metal halide materials introduced a new generation ofthin-filmPVs, due to the excellent characteristics for light harvesting in solar cells. The optical-to-electrical power-conversion efficiency (PCE) of the lead halide perovskite- (CH_3_NH_3_PbI_3_, or MAPbI_3_) based PV device has been increased substantially in recent years [1,2,3,4,5]. The MAPbI_3_ substance favors efficient carrier generation and transport to electrodes. It can absorb sunlight radiation from ultra-violet to infrared region. The electron-hole diffusion length could exceed 1 μm in a tri-halide perovskite absorber [6]. Kim et al. developed the first solid-state lead halide perovskite PV with fluorinated tin oxide (FTO)/TiO_2_/MAPbI_3_/2,2′,7,7′–tetrakis(*N*,*N*-di-*p*-methoxyphenylamino)-,9,9′ spiro-bifluorene (SpiroOMeTAD)/Au nanostructures [7]. Later, Jeng et al. reported the first inverted planar structure of a lead halide perovskite solar cell [8]. Choi et al. studied conjugated polyelectrolytes as the hole-transporting materials (HTM) for inverted-type perovskite solar cells [9]. However, the organic HTM could corrode the electrode materials, which would, in turn, reduce the cell stability. Thus, inorganic type materials, such as CdTe, CuZnSnS, CuZnSnSe, CuZnSnSSe, CuInGaS, CuInGaSe, and CuInGaSSe have become interesting topics for the perovskite solar cells [10,11,12,13]. In addition, FTO should be used in favor overindium-tin-oxide (ITO) as the transparent conducting oxide(TCO), when high-temperature annealing in air is in need for PV device fabrication. This is because the ITO electrical properties can be degraded in the presence of oxygen at relatively high temperature. FTO is more stable after such a high-temperature annealing process. This good transparent conductive layer could significantly decrease photon absorption at the back electrode.

Li et al. reviewed the architectures and deposition methods in standard formation of perovskite and charge-transport layers, and provided an overview on PV device stability [14]. Christians et al. developed tailored interfaces to increase the operational stability of un-encapsulated perovskite solar cells [15]. The long-term stability performance has to be improved for different ingredients to warrant inter-carrier scalability, capability, and system durability. The actual PV devices involved frames, glasses, and other compound materials, which could further challenge the recycling scheme [16]. In addition, metal-selenide had been studied as a carrier-transporting material in perovskite by various sputtering techniques [17,18,19]. This paper has focused on applyingCu_2_ZnSnSe_4_as HTM nano-film, and fabricated by innovative concept to improve Cu-based PV performance. The HTM film should help to maintain perovskite’s open-circuit voltage (Voc), short-circuit current density (J_SC_), fill factor (FF), and stability. An ultra-thin CuZnSnSe HTM (<200 nm) between Mo metal-electrode layer and MAPbI_3_ active absorber layer, would promote carrier transporting, and provide favorable ohmic-contact. The deposition of a good ohmic-contact can reduce interface carrier recombination.

Thin CuInGeSe or CuZnSnS layer could fail to maintain the high efficiency performance. The main reason behind the poor performance was the substantial drop in short-circuit current density [20,21,22]. The Kesterite photovoltaics that applied for Cu_2_ZnSnS_4_, Cu_2_ZnSnSe_4_, and Cu_2_ZnSn(S_1−*x*_Se*_x_*)_4_ emerged as one of the most assured replacements for the chalcopyrite solar cells. The constituent elements are relatively abundant on the earth. They have also demonstrated high absorption coefficients, and direct tunable energy band gaps that would allow effective absorption of photons [23]. The Cu_2_ZnSnSe_4_ HTM film can be deposited in 40~160 nm thickness by magnetron sputtering to achieve well carrier transport. The inorganic CuZnSnSe HTM provides low-cost procedure and material for the applications of perovskite solar cells. 

Chalapathy et al. revealed high-quality CZTS films by high-temperature sulfurization of sputtered Cu/ZnSn/Cu precursor layers [24]. A highly imperfect Cu_2_ZnSnSe_4_ HTM/TCO material interface could be an exhaustion region, leading to high back-electrode contact layer of surface recombination. The selenium diffuses into Mo film’s interface, resulting in possible selenization. The MoSe_2_ film at the interface indicated structural quality for adherence and good electrical contact [25]. The outward diffusion of selenium into Mo strongly depends on the annealing temperature [26]. The reconstructed interfacial layer significantly diminishes series-resistance and enlarges shunt-resistance of the solar cell.

Aqil et al. studied the electrical and morphological appearance of the functional portion of Mo film deposition [27]. A single layer of Mo film with low resistivity and good adhesion has been deposited successfully by the radio-frequency (RF) magnetron sputtering system using appropriate parameters. The nanocrystals could be tuned by the nucleation and growth conditions [28]. The bi-layer Mo films were thus proposed to consist of a porous bottom layer and a dense top layer [29]. It has been possible to control the interfacial MoSe_2_ film thickness during annealing procedure to achieve high solar cell power conversion efficiency [30,31]. The interface of CIGSe/Mo or CZTSe/Mo could improve the structural quality of planar solar cells, and affects the adherence to metal-contacts, providing excellent ohmic-contact at CIGSe/Mo or CZTSe/Mo inter-films. The development of MoSe_2_ could not be formed below 500 °C. The selenization increased strongly on higher annealing temperature, also leading to nano-crystallization orientation. Nevertheless, theCIGSe/Mo or CZTSe/Mo heterojunction could be associated with MoSe_2_ film. It was not an interrelated Schottky-type but was an interconnected ohmic-contact [32,33]. 

Furthermore, ZnS is an ideal inorganic compound to be used as an electron-transporting layer (ETL)for the perovskite PVs [34]. When ZnS dense film is synthesized, it can be transparent, and can be used as a window for visible optics and infrared optics. The film’s nanostructure relies on the deposition procedure or doping element at the growth sequence. In comparison with other complex techniques, this study employed RF (radio frequency) magnetron sputtering process for stable depositing results [35]. The MAPbI_3_/ZnS interface property has been demonstrated by using ultra-violet photoelectron spectra [36]. The ZnS interfacial nano-film could promote the Voc and perovskite PCE performance on PVs. The cascade conduction band architecture effectively decreased the interfacial charge recombination and improved the electron transfer. In addition, Yuan et al. illustrated the reduction in surface defects, which improved charge extraction, extended light response, and expanded ZnS/MAPbI_3_ spectral absorption [37].

Indium-doped zinc oxide (IZO) film only needs a low annealing temperature and can promote the optoelectronic characteristics. It is an amorphous transparent conducting oxide material, and has a downgraded absorption near IR (infra red) region photons, yielding to an enhanced transmission at the bottom layer on solar cells. The IZO material is a potential replacement of traditional TCO that was used as an n-type buffer layer or window transparent conductive oxide layer for photovoltaics. Nevertheless, the fabrication using all sputtering-process methodology, along with the ultra-thin Cu_2_ZnSnSe_4_ HTM, may develop a novel copper-based inorganic HTM for perovskite nanostructured PVs. The results should help to pave the way for the next generation thin-film solar cells and to better protect the global environment.

## 2. Materials and Methods

In this study, bi-layer Mo film was sputtered on FTO glass substrate as a back metal electrode contact layer. The Mo film was prepared by RF magnetron sputtering system using commercial Mo target (Ultimate Materials Technology Co., Miaoli, Taiwan). In this fabrication process, the bottom layer was deposited at a higher Ar flow working pressure, using high-power parameters, and the Ar flow rate and RF power were maintained at 70 sccm and 110 W. On the other hand, the top layer was deposited at a lower Ar flow working pressure, using lower-power parameters, and the Ar flow rate and RF power were maintained at 35 sccm and 55 W for preserving better adhesion. The bi-layer Mo films exhibited both low resistivity and good adhesion. It has been measured that the Mo bi-layer had a film resistivity of 4.37 × 10^−4^ Ω-cm, which was much lower than that of single-layer at 2.2 × 10^−2^ Ω-cm. Each layer had a thickness of ~100 nm. The Mo film also acted as a reflective layer on these multi-layered solar cells. This Mo film that was deposited under high argon pressure would be under tensile stress and adhere successfully with the substrate, but with low conductivity. The deposition by low argon pressure would render compressive stress that had low resistivity but adhered poorly to interface on the substrate.

Moreover, Cu_2_ZnSnSe_4_film was deposited by RF magnetron sputtering using a Cu_2_ZnSnSe_4_ target (Ultimate Materials Technology Co., Miaoli, Taiwan) on bi-layer Mo. The argon flow rate and RF power were maintained at 40 sccm and 70 W, respectively. It was adopted as the HTM layer. It should promote the carrier transporting and result in a beneficial device by providing a conductive ohmic-contact. The ultra-thin Cu_2_ZnSnSe_4_ HTM (<200 nm) surface roughness corresponded to the optical absorber thickness transition, and could affect interface recombination of electrons and electron-holes. The root-mean-square surface roughness was low at ~20 nm, measured by atomic force microscopy. The Cu_2_ZnSnSe_4_ film thickness has been prepared at 40–160 nm approximately. It was then further thermally treated by the annealing temperature at 350, 450, 550, or 650 °C in a tube furnace for about 60 min in order to get magnificent crystallization. 

The MAPbI_3_ film was deposited on grown Cu_2_ZnSnSe_4_ HTM layer and by one-step spin-coating process for the inverted structures of perovskite solar cells. The photovoltaic characteristics would be investigated to reveal the relationships between the properties and structures. The single-step deposition involved the dissolution of PbI_2_ and MAI (CH_3_NH_3_I) in a co-solvent, consisted of equal volumes of dimethyl sulfoxide and gamma-butyrolactone. This perovskite precursor solution was spin-coated using parameters of 1000 and 5000 rpm for 10 and 18 s, respectively, in a nitrogen-filled glove box. The wet film was then quenched by dropping 50 µL of anhydrous toluene at 15 s. Afterwards, the perovskite film was further annealed at 100 °C for 10 min. The MAPbI_3_ thin-film had a thickness of 700 nm approximately. 

Zinc sulfide film is an *n*-type semiconductor material and was adopted as an ETL in this multi-layered nanostructure PVs. It was prepared using a commercial target (Ultimate Materials Technology Co., Miaoli, Taiwan) by RF sputtering system. The Ar flow rate and RF power were controlled at 30 sccm and 50 W, respectively. The ZnS film was about 50 nm in thickness. The film was attributed to not only thinner ETL layer deposition, but also to extract electrons from the MAPbI_3_ active absorber layer. It also required quenching at 100 °C in a tube furnace for about 10 min to achieve the ideal *p-n* junction, crystallization, and better ohmic-contact. The ZnS film could improve the multi-layer structures by alloying, plastic distortion and thermal annealing. It would bond with upper IZO transparent conductive oxide film, while conducting the necessary optical-current passing through, thus enhancing optical-to-electrical conversion performance.

Indium-doped zinc oxide is an n-type semiconductor material and was adopted as a transparent conductive oxide film, deposited by RF magnetron sputtering using a commercially available IZO target (Ultimate Materials Technology Co., Miaoli, Taiwan). The deposition parameters were argon flow rate at 30 sccm and RF power at 50 W. The IZO film had a thickness of 100 nm approximately. The low-temperature and high-mobility amorphous nature rendered excellent characteristics for the ZnS (ETL)/MAPbI_3_/CZTSe (HTM) heterojunction solar cells.

At last, the top Ag metal electrode film was deposited over the IZO *n*-type semiconductor TCO. The Ag metal ingot was prepared on a tungsten metal-boat in the vacuum chamber of an evaporation system. The tungsten boat was connected to an external power supply and provided with a maximum current of 90 A. The Ag metal film had a thickness of ~100 nm. A shadow mask has been adopted to define an active area of 0.5 × 0.2 cm^2^ during the Ag deposition. The nanostructured PV was investigated by X-ray diffraction (XRD) using PANalytical X’Pert Pro DY2840 system (Malvern Panalytical, Almelo, Netherlands) with Cu Kα (λ= 0.1541 nm) radiation. The crystalline surface morphology was studied by scanning electron microscopy (Zeiss Gemini SEM, Jena, Germany). A micro-Raman spectroscopy analysis was employed using Jon-YvonLabRAM system (Horiba-HR800, Kyoto, Japan). The photoluminescence (PL) results were scanned by a fluorescence spectrophotometer (Hitachi, F-7000, Tokyo, Japan). The electron spectroscopy chemical analysis (ESCA) spectra were examined using PHI-5000 system (ULVAC, Versaprobe-II, Kanagawa, Japan). The solar cell PV characteristics were studied by a Keithley 2420 programmable source instrument under 1000 W xenon illumination with a forward scan rate of 0.1 V/s.

## 3. Results and Discussion

**Figure 1:**
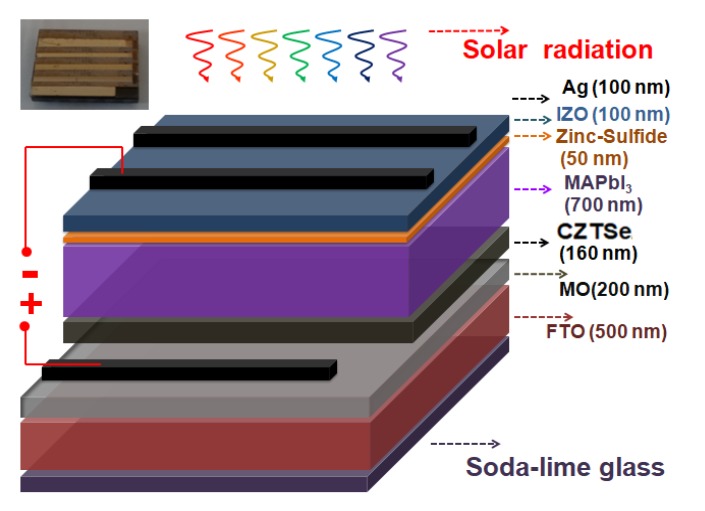
The complete scheme of nanostructured MAPbI_3_ perovskite solar cell with Cu_2_ZnSnSe_4_ HTM layer.

**Figure 2:**
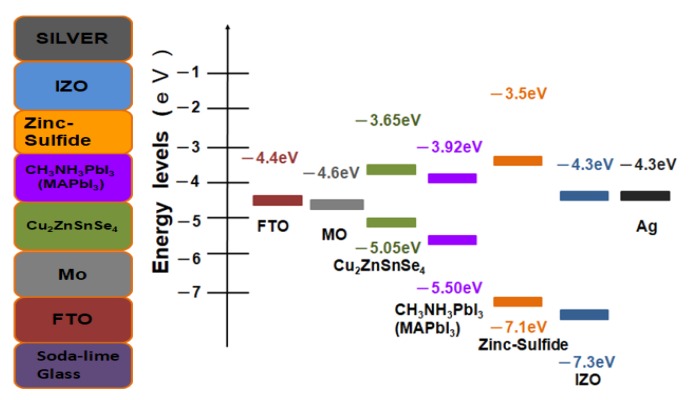
The corresponding energy levels of the planar architecture device of Ag/IZO/ZnS/MAPbI_3_/Cu_2_ZnSnSe_4_/Mo/FTO.

Figure 1 shows the complete scheme of the nanostructured MAPbI_3_ perovskite solar cell device with the Cu_2_ZnSnSe_4_ HTM layer. The corresponding energy levels of the planar architecture device of Ag/IZO/ZnS/MAPbI_3_/Cu_2_ZnSnSe_4_/Mo/FTO are illustrated in Figure 2. The ultra-thin Cu_2_ZnSnSe_4_ HTM has been deposited between the bi-layer Mo metal-electrode and the MAPbI_3_ active absorber layer to improve carrier transporting. The energy level diagram indicated that it is a heterojunction planar photovoltaic solar cell.

**Figure 3:**
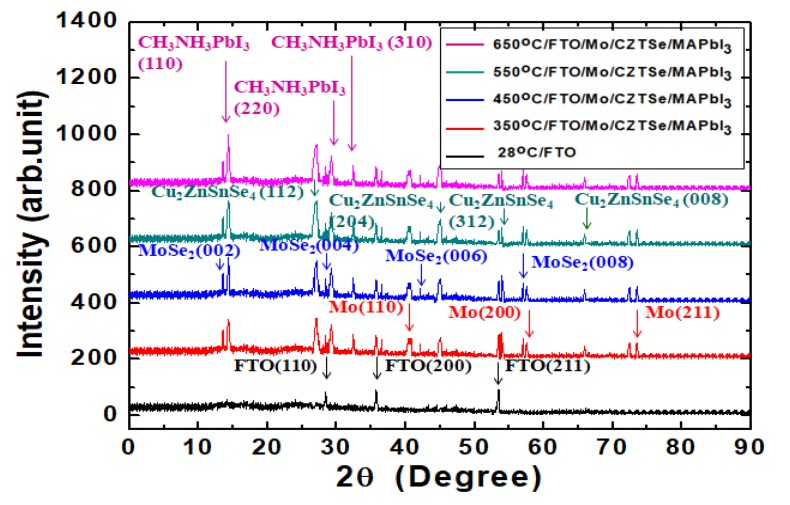
XRD diffraction pattern results of the MAPbI_3_/Cu_2_ZnSnSe_4_/Mo/FTO nanostructures on glass substrate after the various annealing temperatures.

Figure 3 shows the XRD diffraction pattern results of the MAPbI_3_/Cu_2_ZnSnSe_4_/Mo/FTO nanostructures on glass substrate after the various annealing temperatures [3,21,31]. The MAPbI_3_ film’s nano-crystal was illustrated by one main crystal plane (110) corresponding to the 2θ diffraction peak at ~14.3°. The 2θ full-width at half-maximum (FWHM) was reduced from 0.39° to 0.28° when the annealing temperature was increased from 350 °C to 650 °C. Other MAPbI_3_ crystal planes involved (220) at ~29.2° and (310) at ~32.4°. When the annealing temperature of the Cu_2_ZnSnSe_4_ HTM film under the MAPbI_3_ film was increased, the FWHM of the Cu_2_ZnSnSe_4_ HTM film’s nano-crystal was also improved. Its nano-crystal was illustrated by the main crystal plane (112) corresponding to the 2θ diffraction peak at ~27.1°. Its FWHM was reduced from 0.64° to 0.51° when the annealing temperature was increased from 350 to 650 °C. Other Cu_2_ZnSnSe_4_ crystal planes could be illustrated by (204) at 2θ diffraction peak of ~45.1°, (312) at ~53.8°, and (008) at 65.8°. The Mo film’s nano-crystal was illustrated by the main crystal plane (110) at the 2θ diffraction peak at ~40.5°. Its FWHM was reduced from 0.98° to 0.83° when the annealing temperature was increased from 350 to 650 °C. Other Mo film’s crystal planes included (200) at ~58.6°, and (211) at ~73.5°. The smaller FWHM demonstrated MoSe_2_ nano-crystal and it could be illustrated by the main crystal plane (002) at the 2θ diffraction peak at ~13.5°. The FWHM was reduced from 0.13° to 0.10° when the annealing temperature was increased from 350 to 650 °C. Other MoSe_2_ crystal planes included (004) at ~28.2°, (006) at ~42.7°, and (008) at ~57.5°. The Cu_2_ZnSnSe_4_ HTM film has been annealed at 350, 450, 550, and 650 °C, respectively. As a result, the film’s crystal quality was improved following the increased annealing temperature. Interestingly, the crystallization of MAPbI_3_ active absorber layer was also preceded by the same annealing temperature. It has been noted that when one side of a heterojunction is much more heavily doped than the other side, the junction is nearly a one-sided heterojunction. To form a good quality heterojunction, the difference between the neighboring semiconductors’ lattice constants should be small in order to minimize the density of interface states. The difference in electron hole affinity between two different materials should be small to minimize band discontinuity, and thermal expansion coefficients should be close as well. MAPbI_3_ film and Cu_2_ZnSnSe_4_ HTM film were likely annealed and sintered altogether. 

Furthermore, their energy band gaps were similar for trapping light simultaneously. It would be beneficial to constitute pairs of excited electrons and associated electron holes. Eventually, the carriers could increase the optical-electronic power-conversion efficiency and optical-current associated in the photovoltaic cell’s multi-layer nanostructures.

**Figure 4:**
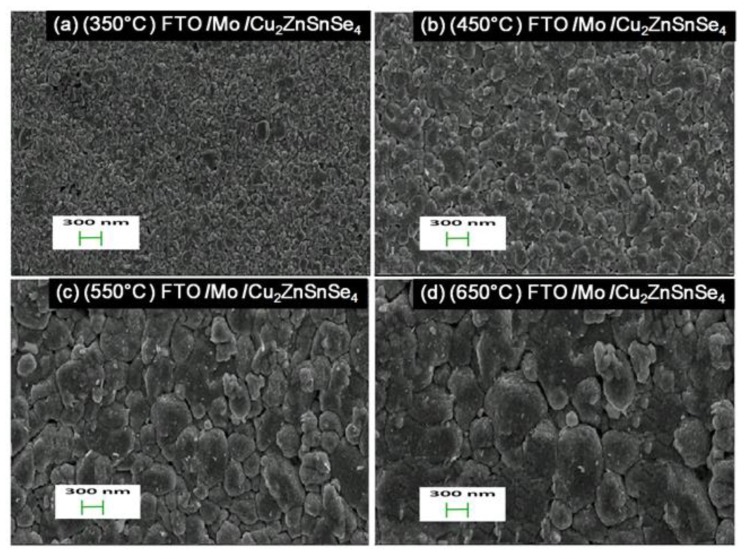
Top-view SEM micrographs of theCu_2_ZnSnSe_4_ HTM layers after the various annealing temperatures of: (**a**) 350 °C; (**b**) 450 °C; (**c**) 550 °C; (**d**) 650 °C.

Figure 4 shows the top-view SEM micrographs of the surface morphology of Cu_2_ZnSnSe_4_ HTM layers after the various annealing temperatures. The magnetron sputtered film provided full surface coverage and was composed of small crystal grains ranging from tens of nm to one μm in size. After its deposition, the bi-layer Mo film became indiscernible. However, the Cu_2_ZnSnSe_4_ nano-crystal grainsize was significantly enlarged with the increased annealing temperature. For the 350 °C-annealed sample, it exhibited relatively small crystal grains from tens to two hundred nm. It contained some pinhole-type of defects. Figure 4b showed Cu_2_ZnSnSe_4_ film surface appearance with tens to four hundred nm in grain sizefor 450 °C. Figure 4c showed film surface with tens to six hundred nm in the grain sizefor 550 °C. It also contained less pinholes at the same magnification. Figure 4d showed the Cu_2_ZnSnSe_4_ film surface morphology for 650 °C. It demonstrated better crystallization, with crystal grains as large as one μm in size. Much less pinholes could be found. Thus, the higher annealing temperature achieved high-quality surface HTM films. Additionally, the Cu_2_ZnSnSe_4_ HTM film’s hole mobility was increased from 15.1 cm^2^/(V s) to 29 cm^2^/(V s), while the annealing temperature was increased from 350 to 650 °C. The higher carrier mobility would help to reduce the device series-resistance, and improve performance for the nanostructured photovoltaic cells.

**Figure 5:**
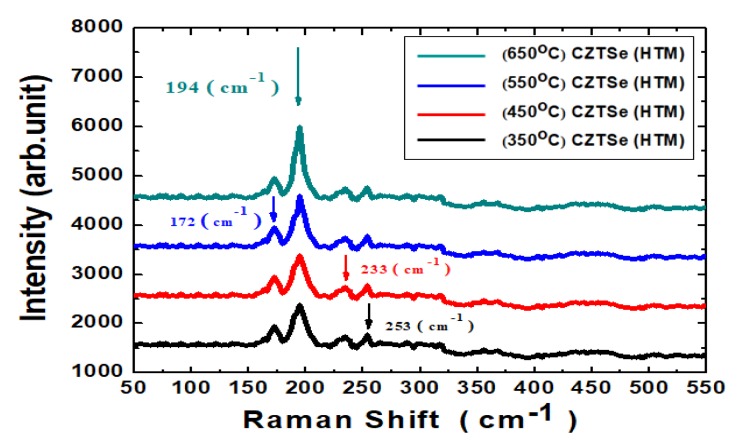
The compositional dependence of Raman spectra of the Cu_2_ZnSnSe_4_ HTM nano-films. The arrows point towards the corresponding wave numbers.

Figure 5 shows the compositional dependence of Raman spectra of the Cu_2_ZnSnSe_4_ HTM nano-films after the various annealing temperature treatments. In this fabrication, Cu_2_ZnSnSe_4_ HTM film exhibited dominant spectra with intense Raman scattering main peak at 194 cm^−1^ correlating with the optical phonon mode. Its intensity increased slightly with the increased thermal annealing temperature. Other Raman scattering peak intensities were found at 233 and 253 cm^−1^. The Cu_2_ZnSnSe_4_ HTM crystal orientation could be found from polarization of Raman-scattered light with respect to the laser light, if the crystal structure’s point group could be known. 

**Figure 6:**
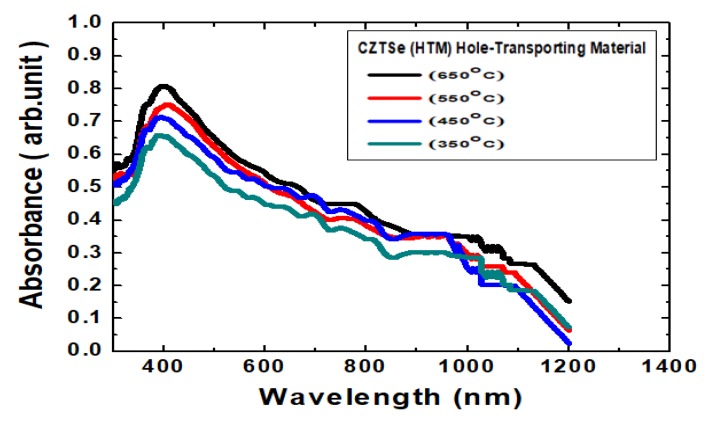
The absorbance spectra of Cu_2_ZnSnSe_4_ HTM nano-films after the various annealing temperatures.

**Figure 7:**
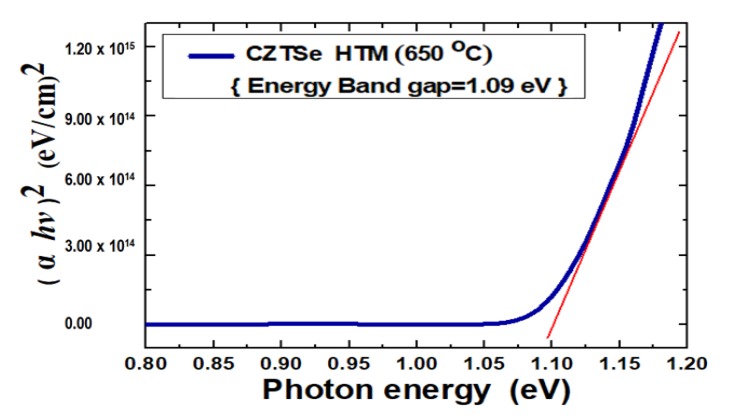
Graph of *hν* versus (*αhν*)^2^ for measuring the optical energy bandgap of Cu_2_ZnSnSe_4_. It was determined by extrapolating the straight line portion to the photon energy axis.

Figure 6 shows the measurement results of the absorbance spectra of Cu_2_ZnSnSe_4_ HTM nano-films after the various annealing temperatures. The optical absorption properties of Cu_2_ZnSnSe_4_ in the visible region and near-infrared region are associated with electronic transitions and are also useful in comprehending electronic band conformations of semiconducting films. The optical spectra were recorded using a UV (ultraviolet) spectrophotometer at the wavelength range from 300 to 1200 nm. It was observed that the absorbance intensity increased with the increased annealing temperature. This was presumably caused by free-carrier absorption corresponding to conductivity. These absorption spectra illustrated that all Cu_2_ZnSnSe_4_ nano-films absorb over the entire visible region of electromagnetic waves. The absorption spectra data were analyzed following a classical equation for near edge optical absorption of semiconductors: *αhν* = A(*hν* − *E_g_*)*^n^*, where *α* is absorption coefficient, *hν* is photon energy, *E_g_* is energy band gap, A is constant, *n* can have values of 1/2, 2, 3/2 and 3 for allowed direct, allowed indirect, forbidden direct and forbidden indirect transitions, separately. The Cu_2_ZnSnSe_4_ energy band gap was determined by plotting a graph of *hv* versus (*αhv*)^2^, for the direct band gap. The energy band gap was designated by extrapolating the straight line portion to the energy axis, whose intercept to the *x*-axis should give the optical energy band gap [38]. An example graph for Cu_2_ZnSnSe_4_annealed at 650 °C is provided in Figure 7, and the calculation result of energy band gap has been 1.07–1.1 eV for the ultra-thin Cu_2_ZnSnSe_4_ HTM which was deposited on bi-layer Mo/FTO glass substrate. It should promote a valence electron bound to an atom to a conduction electron. Such electrons then move freely within the HTM and become carriers that can conduct current.

**Figure 8:**
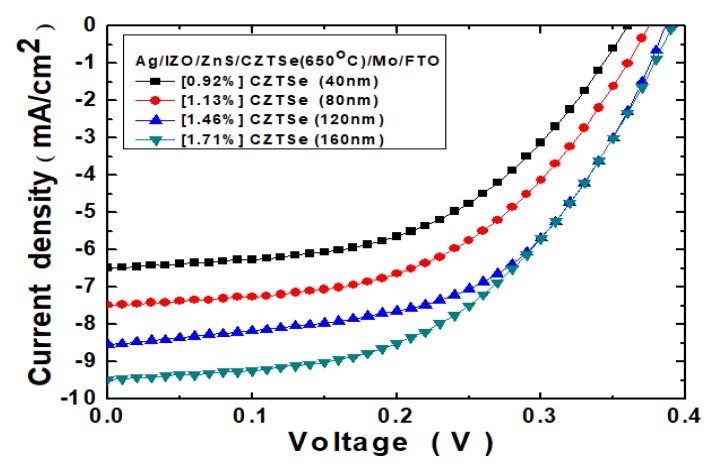
The *J*–*V* curves of Ag/IZO/ZnS/Cu_2_ZnSnSe_4_/Mo/FTO nanostructured solar cells, under 100 mW/cm^2^ illumination. The Cu_2_ZnSnSe_4_ HTM layer thickness has been varied at 40–160 nm.

Figure 8 displays the graphic current-density voltage (*J*–*V*) curves of Ag/IZO/ZnS/Cu_2_ZnSnSe_4_/Mo/FTO nanostructured solar cells. The Cu_2_ZnSnSe_4_ HTM layer thickness has been varied at 40~160 nm, and the thermal annealing temperature was all at 650 °C. Additionally, Table 1 summarizes the PV characteristic parameters of these nanostructured solar cells, without MAPbI_3_ perovskite, under 100 mW/cm^2^ illumination (air mass, AM1.5G). It has been evidenced that the open-circuit voltage increased from 0.36 to 0.39 V, following the increased HTM layer thickness from 40 to 160 nm. The device short-circuit current was also enlarged from 6.47 to 9.46 mA/cm^2^. The device fill factor value would be amplified from 39.5% to 46.3%. The PV device power-conversion efficiency value was slightly increased from 0.92% to 1.71%, and the output power *P*_max_ value was enhanced from 0.09 to 0.17 mW. Additionally, the Cu_2_ZnSnSe_4_ film alone could not absorb enough photons, so that the PV cells exhibited poor PCE performance in the illustration.

**Figure 9:**
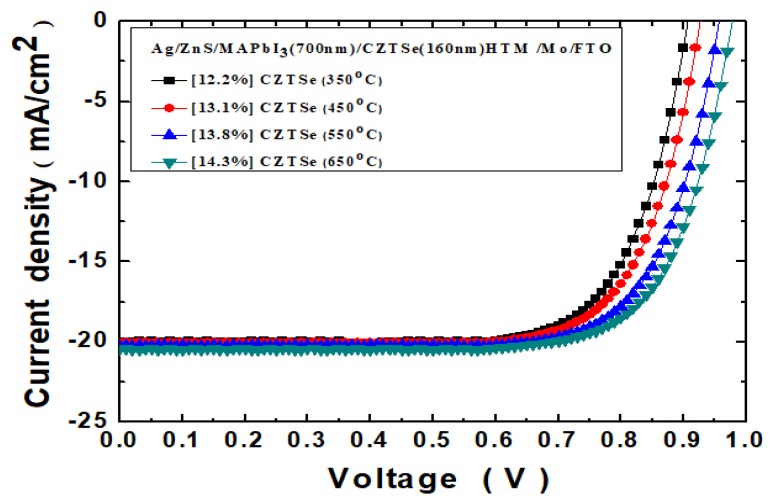
The *J*–*V* curves of Ag/ZnS/MAPbI_3_/Cu_2_ZnSnSe_4_/Mo/FTO nanostructured solar cells, under 100 mW/cm^2^ illumination. The Cu_2_ZnSnSe_4_HTM has been thermally treated at the various annealing temperature 350–650 °C.

Figure 9 displays the graphic *J*–*V* curves of Ag/ZnS/MAPbI_3_/Cu_2_ZnSnSe_4_/Mo/FTO nanostructured solar cells, with the MAPbI_3_ perovskite nanostructures on bi-layer Mo. The Cu_2_ZnSnSe_4_ HTM layer thickness has been fixed at 160 nm, but was thermally treated at the various annealing temperature 350–650 °C. The measurements were, again, under 100 mW/cm^2^ illumination. Table 2 lists the derived PV characteristic parameters of the Ag/ZnS/MAPbI_3_/Cu_2_ZnSnSe_4_/Mo/FTO nanostructured solar cells. It has been clearly evidenced with significant improvements in all the parameters. The open-circuit voltage was increased from 0.89 to 0.98 V following the increased annealing temperature from 350 to 650 °C. The short-circuit current was also expanded from 19.9 to 20.4 mA/cm^2^. The device fill factor value could be increased from 68.6% to 71.3%. The PV device power-conversion efficiency value was strengthened from 12.2% to 14.3%, and the output power *P_max_* value was enhanced from 1.22 to 1.43 mW.

**Figure 10:**
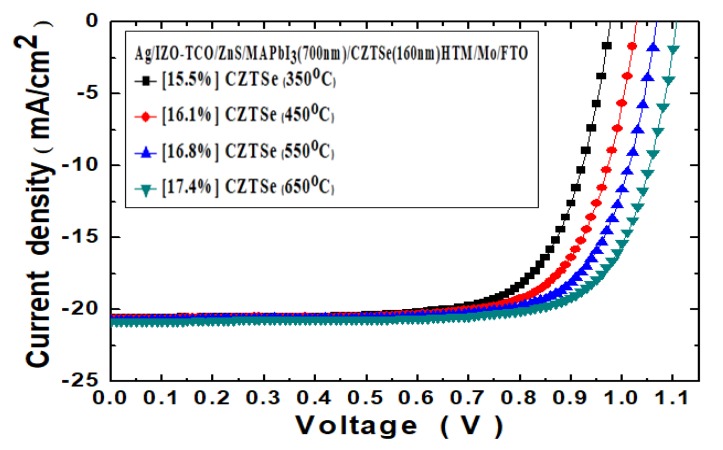
The *J*–*V* curves of Ag/IZO/ZnS/MAPbI_3_/Cu_2_ZnSnSe_4_/Mo/FTO nanostructured solar cells, under 100 mW/cm^2^ illumination. The IZO layer thickness has been 100 nm.

In addition, Figure 10 displays the *J*–*V* curves of the Ag/IZO/ZnS/MAPbI_3_/Cu_2_ZnSnSe_4_/Mo/FTO nanostructured solar cells at the various HTM thermal annealing temperatures under 100 mW/cm^2^ illumination. The TCO layer in thickness of 100 nm IZO film has been inserted between the ZnS ETL and top Ag electrode. Table 3 presents the derived PV characteristic parameters of the Ag/IZO/ZnS/MAPbI_3_/Cu_2_ZnSnSe_4_/Mo/FTO nanostructured solar cells. Furthermore, it has been clearly evidenced with enhancements in all the parameters. The open-circuit voltage was amplified from 0.97 to 1.10 V, following the annealing temperature that was increased from 350 to 650 °C. The short-circuit current was slightly increased from 20.5 to 20.8 mA/cm^2^. The device fill factor value was slightly diminished to 76.3%. The PV device power-conversion efficiency value was further increased from 15.5% to 17.4%. Additionally, the device series-resistance was decreased from 20.2 to 17.1 Ω., and the device output power *P_max_* value could be enhanced from 1.55 to 1.74 mW. 

**Figure 11:**
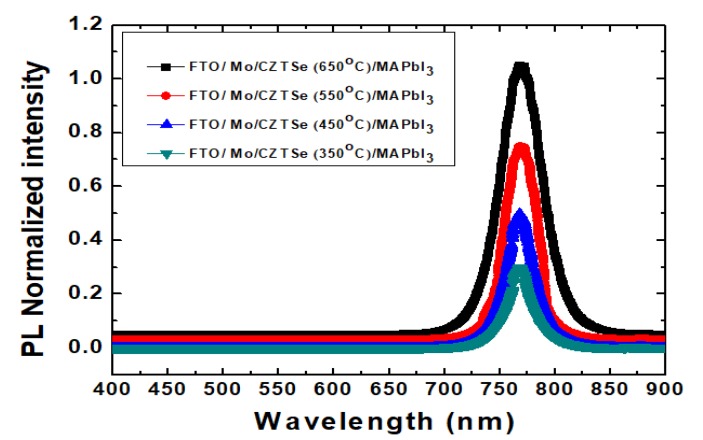
PL spectra of MAPbI_3_ perovskite nano-films on Cu_2_ZnSnSe_4_/Mo/FTO following the various thermal annealing temperatures.

Figure 11 shows the PL spectral measurement results of MAPbI_3_ perovskite nano-films onCu_2_ZnSnSe_4_/Mo/FTO following the various thermal annealing temperatures. The spectra were examined by fluorescence spectrophotometer. It has been clearly evidenced with one main peak at the wavelength of ~768 nm. The intensity was also enhanced by the increased annealing temperature. The intensity of PL spectrum is relative to lifetime of the injected electrons and electron-holes combined to form excitons. An exciton indicates a mobile energy constitution by an excited electron and a relative electron-hole. Anincrease in the number of excitons could simultaneously increase the electron/electron-hole recombination, thus the PL intensity.

**Figure 12:**
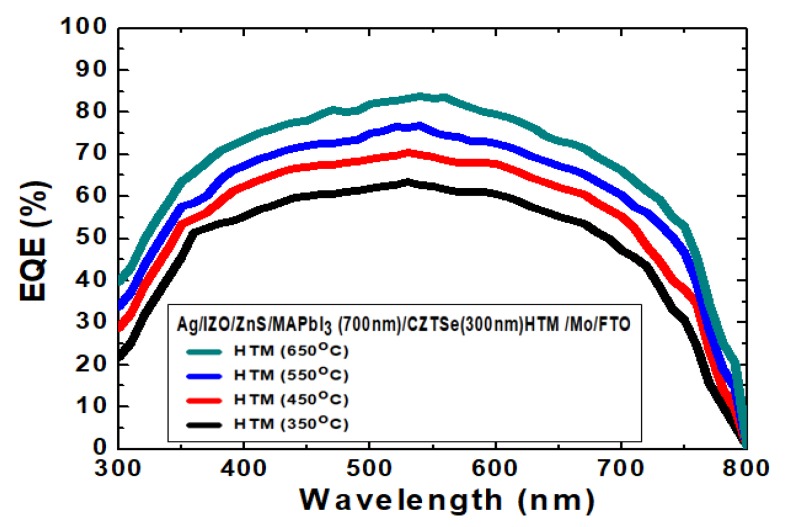
The EQE spectra based on Ag/IZO/ZnS/MAPbI_3_/Cu_2_ZnSnSe_4_/Mo/FTO nanostructured solar cells.

Figure 12 displays the external quantum efficiency (EQE) spectrum measurement results based on Ag/IZO/ZnS/MAPbI_3_/Cu_2_ZnSnSe_4_/Mo/FTO nanostructured solar cells after the various annealing temperatures. The EQE curves for the planar solar cells have been well increased in the wavelength range of 300~800 nm from the 350 °C to 650 °C samples. Among the results of these measurements, the maximum EQE value reached nearly 85% at 550 nm for the 650 °C sample, as compared to 63% for the 350 °C sample. The higher EQE value could suggest a reduction of recombination centers. Planar solar cells involved translational electrical field distribution. It was determined entirely by 1-dimensional resonance.

**Figure 13:**
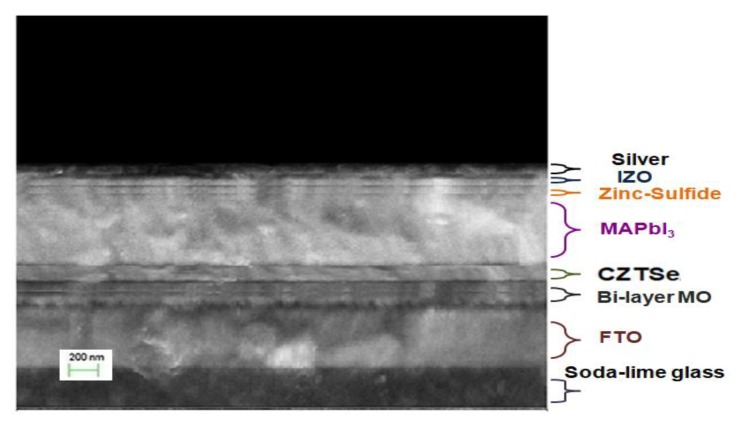
SEM cross-sectional micrograph of the Ag/IZO/ZnS/MAPbI_3_/Cu_2_ZnSnSe_4_/Mo/FTO nanostructures on glass substrate.

**Figure 14:**
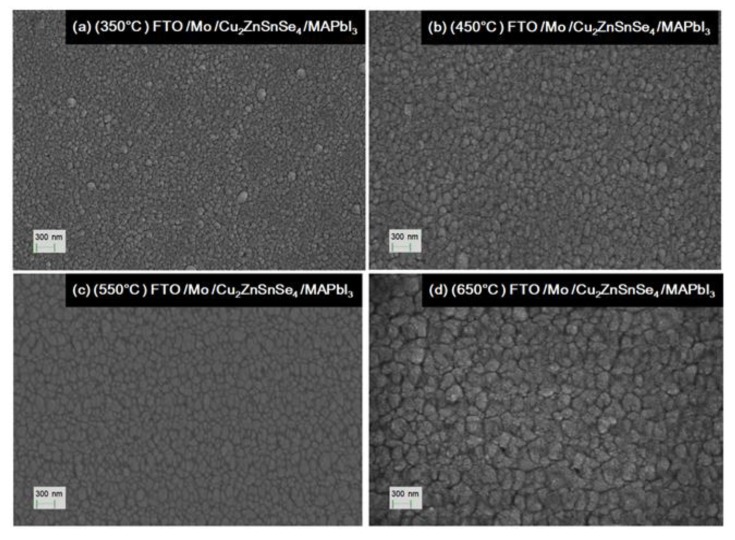
SEM micrographs of MAPbI_3_ perovskite films on Cu_2_ZnSnSe_4_/Mo/FTO following the various thermal annealing temperatures: (**a**) 350 °C; (**b**) 450 °C; (**c**) 550 °C; (**d**) 650 °C.

Figure 13 shows the cross-sectional SEM micrograph of the Ag/IZO/ZnS/MAPbI_3_/Cu_2_ZnSnSe_4_/Mo/FTO nanostructures on glass substrate. This sample has been annealed at 650 °C. The MAPbI_3_ perovskite solar cell was evidenced with a glossy cross-section, contained few pinholes and micro-crack type of defects. The planar solar cell had uniform layer distribution through the magnetron sputtering technique and the spin-coating process. The thickness of each constituent layer could be clearly identified. Consequently, the nano-crystals were of high quality from this fabrication process, and the PV cell characteristics should be warranted in this study. In addition, Figure 14 shows the SEM micrographs of MAPbI_3_ perovskite films on Cu_2_ZnSnSe_4_/Mo/FTO following the various thermal annealing temperatures. The crystal grains exhibited good surface coverage and were significantly increased in size with the increased annealing temperature.

**Figure 15:**
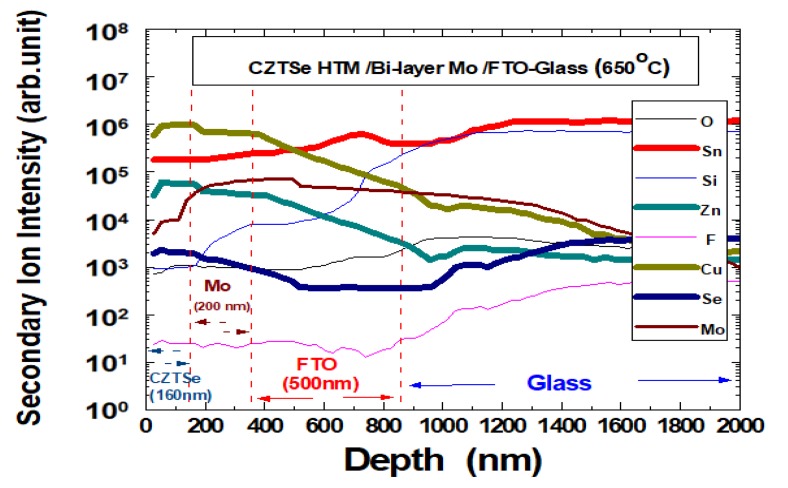
SIMS depth profile of the Cu_2_ZnSnSe_4_ HTM nano-film grown on bi-layer Mo/FTO glass.

Figure 15 demonstrates the secondary ion mass spectrometry (SIMS) depth profile of the Cu_2_ZnSnSe_4_ HTM nano-film grown on bi-layer Mo/FTO glass substrate, using ESCA PHI-5000 system (Ulvac-PHI, Kanagawa, Japan). This sample has been annealed at 650 °C. The addition of Cu-based Cu_2_ZnSnSe_4_ material, including selenide and MoSe_2_ inter-film, had beneficial effect on the power conversion efficiency. The SIMS analysis provided quantitative depth profiling with good depth resolution. These features became essential to characterize the ultra-thin Cu_2_ZnSnSe_4_ HTM solar cells, where possible variations on structure or composition could lead to significant changes. In this study, Cu_2_ZnSnSe_4_ HTM film was prepared by magnetron sputtering with 160 nm in thickness, and bi-layer Mo back contact layer at about 200 nm on FTO glass substrate, all well evidenced in the depth profile. It has been noted that the use of a large collection area would provide a more representative sampling of the results. Nevertheless, the SIMS depth profile can be reconstructed after the deposition analysis to provide compositional changes at different locations.

## 4. Conclusions

In summary, one-step magnetron sputtered Cu_2_ZnSnSe_4_ nano-films have been successfully applied as novel Cu-based inorganic HTM for MAPbI_3_ perovskite nanostructured photovoltaics. The adequate control in nano-film quality significantly improved the PV characteristic parameters of Ag/ZnS/MAPbI_3_/Cu_2_ZnSnSe_4_/Mo/FTO nanostructured solar cells. When the Cu_2_ZnSnSe_4_ HTM thickness was designed at 160 nm, and the thermal annealing temperature wasat 650 °C, its open-circuit voltage was increased to 0.98 V, and short-circuit current was increased to 20.4 mA/cm^2^. The device fill factor value was increased to 71.3%, the power-conversion efficiency value was elevated to 14.3%, and the output power *P_max_* value was enhanced to 1.43 mW. Furthermore, the additional inclusion of transparent conductive IZO could further enhance the PV characteristic parameters of the Ag/IZO/ZnS/MAPbI_3_/Cu_2_ZnSnSe_4_/Mo/FTO nanostructured solar cells. The open-circuit voltage was enhanced to 1.10 V, and the short-circuit current was increased to 20.8 mA/cm^2^. The device fill factor value was improved to 76.3%, the power-conversion efficiency value was increased to 17.4%, the device series-resistance was decreased to 17.1 Ω., and the device output power *P_max_* value could be enhanced to 1.74 mW. Therefore, the Cu_2_ZnSnSe_4_ HTM used in this study would help the development of perovskite PV technology.

## Figures and Tables

**Table 1:** The PV characteristic parameters of the Ag/IZO/ZnS/Cu_2_ZnSnSe_4_/Mo/FTO nanostructured solar cells.

Cu_2_ZnSnSe_4_ HTM Thickness (nm)	*V_OC_* (V)	*J_SC_* (mA/cm^2^)	*FF* (%)	*Eff* (%)	*P*_max_ (mW)
40	0.36	6.47	39.5	0.92	0.09
80	0.37	7.47	40.9	1.13	0.11
120	0.38	8.52	45.1	1.46	0.15
160	0.39	9.46	46.3	1.71	0.17

**Table 2:** The PV characteristic parameters of the Ag/ZnS/MAPbI_3_/Cu_2_ZnSnSe_4_/Mo/FTO nanostructured solar cells.

Cu_2_ZnSnSe_4_ HTM Annealing Temperature (°C)	*V_OC_* (V)	*J_SC_* (mA/cm^2^)	*FF* (%)	*Eff* (%)	*P*_max_ (mW)
350	0.89	19.9	68.6	12.2	1.22
450	0.92	20.1	70.9	13.1	1.31
550	0.95	20.2	71.2	13.8	1.38
650	0.98	20.4	71.3	14.3	1.43

**Table 3:** The PV characteristic parameters of the Ag/IZO/ZnS/MAPbI_3_/Cu_2_ZnSnSe_4_/Mo/FTO nanostructured solar cells.

Cu_2_ZnSnSe_4_ HTM Annealing Temperature (°C)	*V_OC_* (V)	*J_SC_* (mA/cm^2^)	*FF* (%)	*Eff* (%)	*R_s_* (Ω)	*P*_max_ (mW)
350	0.97	20.5	77.6	15.5	20.2	1.55
450	1.02	20.6	76.4	16.1	19.7	1.61
550	1.06	20.7	76.3	16.8	18.2	1.68
650	1.10	20.8	76.3	17.4	17.1	1.74

## References

[1] Safari Z., Zarandi M.B., Giuri A., Bisconti F., Carallo S., Listorti A., Corcione C.E., Nateghi M.R., Rizzo A., Colella S. (2019). Optimizing the interface between hole transporting material and nanocomposite for highly efficient perovskite solar cells. Nanomaterials.

[2] Jeon N.J., Noh J.H., Yang W.S., Kim Y.C., Ryu S., Seo J., Seok S.I. (2015). Compositional engineering of perovskite materials for high-performance solar cells. Nature.

[3] Wang T., Zhang H., Hou S., Zhang Y., Li Q., Zhang Z., Gao H., Mao Y. (2019). Facile synthesis of methylammonium lead iodide perovskite with controllable morphologies with enhanced luminescence performance. Nanomaterials.

[4] Masood M.T., Qudsia S., Hadadian M., Weinberger C., Nyman M., Ahlang C., Dahlstrom S., Liu M., Vivo P., Osterbacka R. (2020). Investigation of well-defined pinholes in TiO_2_ electron selective layers used in planar heterojunction perovskites solar cells. Nanomaterials.

[5] Bresolin B.M., Hammouda S.B., Sillanpaa M. (2020). An emerging visible-light organic-inorganic hybrid perovskite for photocatalytic applications. Nanomaterials.

[6] Stranks S.D., Eperon G.E., Grancini G., Menelaou C., Alcocer M.J., Leijtens T., Herz L.M., Petrozza A., Snaith H.J. (2013). Electron-hole diffusion lengths exceeding 1 micrometer in an organometal trihalide perovskite absorber. Science.

[7] Kim H.S., Lee C.R., Im J.H., Lee K.B., Moehl T., Marchioro A., Moon S.J., Humphry-Baker R., Yum J.H., Moser J.E. (2012). Lead iodide perovskite sensitized all-solid-state submicron thin film mesoscopic solar cell with efficiency exceeding 9%. Sci. Rep..

[8] Jeng J.Y., Chiang Y.F., Lee M.H., Peng S.R., Guo T.F., Chen P., Wen T.C. (2013). CH_3_NH_3_PbI_3_ perovskite/fullerene planar-heterojunction hybrid solar cells. Adv. Mater..

[9] Choi H., Mai C.K., Kim H.B., Jeong J., Song S., Bazan G.C., Kim J.Y., Heeger A.J. (2015). Conjugated polyelectrolyte hole transport layer for inverted-type perovskite solar cells. Nat. Commun..

[10] Battaglia C., Cuevas A., De Wolf S. (2016). High-efficiency crystalline silicon solar cells: Status and perspectives. Energy Environ. Sci..

[11] Wu Q., Xue C., Li Y., Zhou P., Liu W., Zhu J., Dai S., Zhu C., Yang S. (2015). Kesterite Cu_2_ZnSnS_4_ as a low-cost inorganic hole-transporting material for high-efficiency perovskite solar cells. ACS Appl. Mater. Interfaces.

[12] Ramanujam J., Singh U.P. (2017). Copper indium gallium selenide based solar cells—A review. Energy Environ. Sci..

[13] Van Lare C., Yin G., Polman A., Schmid M. (2015). Light coupling and trapping in ultrathin Cu(In,Ga)Se_2_ solar cells using dielectric scattering patterns. ACS Nano.

[14] Li Z., Klein T.R., Kim D.H., Yang M., Berry J.J., Van Hest M., Zhu K. (2018). Scalable fabrication of perovskite solar cells. Nat. Rev. Mater..

[15] Christians J.A., Schulz P., Tinkham J.S., Schloemer T.H., Harvey S.P., De Villers B.J., Sellinger A., Berry J.J., Luther J.M. (2017). Tailored interfaces of unencapsulated perovskite solar cells for >1000 h operational stability. Nat. Energy.

[16] Jackson P., Wuerz R., Hariskos D., Lotter E., Witte W., Powalla M. (2016). Effects of heavy alkali elements in Cu(In,Ga)Se_2_ solar cells with efficiencies up to 22.6%. Phys. Status Solidi RRL.

[17] Wen X., Chen C., Lu S., Li K., Kondrotas R., Zhao Y., Chen W., Gao L., Wang C., Zhang J. (2018). Vapor transport deposition of antimony selenide thin film solar cells with 7.6% efficiency. Nat. Commun..

[18] Sapkota Y.R., Mazumdar D. (2018). Bulk transport properties of bismuth selenide thin films grown by magnetron sputtering approaching the two-dimensional limit. J. Appl. Phys..

[19] Ong K.H., Agileswari R., Maniscalco B., Arnou P., Kumar C.C., Bowers J.W., Marsadek M.B. (2018). Review on substrate and molybdenum back contact in CIGS thin film solar cell. Int. J. Photoenergy.

[20] Khoshsirat N., Ali F., Tiong V.T., Amjadipour M., Wang H., Shafiei M., Motta N. (2018). Optimization of Mo/Cr bilayer back contacts for thin-film solar cells. Beilstein J. Nanotechnol..

[21] Dalapati G.K., Zhuk S., Masudy-Panah S., Kushwaha A., Seng H.L., Chellappan V., Suresh V., Su Z., Batabyal S.K., Tan C.C. (2017). Impact of molybdenum out diffusion and interface quality on the performance of sputter grown CZTS based solar cells. Sci. Rep..

[22] Borri C., Calisi N., Galvanetto E., Falsini N., Biccari F., Vinattieri A., Cucinotta G., Caporali S. (2020). First proof-of-principle of inorganic lead halide perovskites deposition by magnetron-sputtering. Nanomaterials.

[23] Yin L., Cheng G., Feng Y., Li Z., Yang C., Xiao X. (2015). Limitation factors for the performance of kesterite Cu_2_ZnSnS_4_ thin film solar cells studied by defect characterization. RSC Adv..

[24] Chalapathy R.B.V., Jung G.S., Ahn B.T. (2011). Fabrication of Cu_2_ZnSnS_4_ films by sulfurization of Cu/ZnSn/Cu precursor layers in sulfur atmosphere for solar cells. Sol. Energy Mater. Sol. Cells.

[25] Yin G., Knight M.W., Van Lare M.C., Garcia M.M.S., Polman A., Schmid M. (2017). Optoelectronic enhancement of ultrathin CuIn_1−x_Ga_x_Se_2_ solar cells by nanophotonic contacts. Adv. Opt. Mater..

[26] Khojier K., Mehr M.R., Savaloni H. (2013). Annealing temperature effect on the mechanical and tribological properties of molybdenum nitride thin films. J. Nanostruct. Chem..

[27] Aqil M.M., Azam M.A., Aziz M.F., Latif R. (2017). Deposition and characterization of molybdenum thin film using direct current magnetron and atomic force microscopy. J. Nanotechnol..

[28] Chen C., Zhang L., Shi T., Liao G., Tang Z. (2019). Controllable synthesis of all inorganic lead halide perovskite nanocrystals with various appearances in multiligand reaction system. Nanomaterials.

[29] Huang P.C., Sung C.C., Chen J.H., Huang C.H., Hsu C.Y. (2017). The optimization of a Mo bi-layer and its application in Cu(In,Ga)Se_2_ solar cells. Appl. Surf. Sci..

[30] Shin B., Zhu Y., Bojarczuk N.A., Chey S.J., Guha S. (2012). Control of an interfacial MoSe_2_ layer in Cu_2_ZnSnSe_4_ thin film solar cells: 8.9% power conversion efficiency with a TiN diffusion barrier. Appl. Phys. Lett..

[31] Lin Y.C., Liu K.T., Hsu H.R. (2018). A comparative investigation of secondary phases and MoSe_2_ in Cu_2_ZnSnSe_4_ solar cells: Effect of Zn/Sn ratio. J. Alloy. Compd..

[32] Nishimura T., Sugiura H., Nakada K., Yamada A. (2018). Characterization of interface between accurately controlled Cu-deficient layer and Cu(In,Ga)Se_2_ absorber for Cu(In,Ga)Se_2_ solar cells. Phys. Status Solidi RRL.

[33] Zhang X., Kobayashi M., Yamada A. (2017). Comparison of Ag(In,Ga)Se_2_/Mo and Cu(In,Ga)Se_2_/Mo interfaces in solar cells. ACS Appl. Mater. Interfaces.

[34] Zheng E., Wang Y., Song J., Wang X.F., Tian W., Chen G., Miyasaka T. (2018). ZnO/ZnS core-shell composites for low-temperature-processed perovskite solar cells. J. Energy Chem..

[35] Ke W., Stoumpos C.C., Logsdon J.L., Wasielewski M.R., Yan Y., Fang G., Kanatzidis M.G. (2016). TiO_2_-ZnS cascade electron transport layer for efficient formamidinium tin iodide perovskite solar cells. J. Am. Chem. Soc..

[36] Liu J., Gao C., Luo L., Ye Q., He X., Ouyang L., Guo X., Zhuang D., Liao C., Mei J. (2015). Low-temperature, solution processed metal sulfide as an electron transport layer for efficient planar perovskite solar cells. J. Mater. Chem. A.

[37] Yuan H., Zhao Y., Duan J., Wang Y., Yang X., Tang Q. (2018). All-inorganic CsPbBr3 perovskite solar cell with 10.26% efficiency by spectra engineering. J. Mater. Chem. A.

[38] Shyju T.S., Anandhi S., Suriakarthick R., Gopalakrishnan R., Kuppusami P. (2015). Mechanosynthesis, deposition and characterization of CZTS and CZTSe materials for solar cell applications. J. Solid State Chem..

